# Dynamic event-triggered delay compensation control for networked predictive control systems with random delay

**DOI:** 10.1038/s41598-023-46753-1

**Published:** 2023-11-16

**Authors:** Ji Zhang

**Affiliations:** College of Electrical Engineering and Automation, Shandong University of Science and Technology, Qingdao, China

**Keywords:** Electrical and electronic engineering, Information theory and computation, Computational science

## Abstract

This paper aims to investigate the dynamic event-triggered control problem for networked predictive control systems with random delays and disturbance. First, a discrete-time dynamic event-triggered control scheme, in which sensor information is only updated when it is necessary, is presented. Next, the systems are modeled as a time-delay singular Markovian jump systems with time-varying switching. Then, a dynamic event-triggered delay compensation control strategy is proposed. Sufficient conditions guaranteeing the asymptotically stable are derived based on the Lyapunov–Krasovskii functional method together with the linear matrix inequality (LMI) technique. Finally, simulation results verify the effectiveness of the proposed strategy.

## Introduction

Networked control systems (NCSs) have garnered research attention and produced a number of exceptional works over the past 20 years as a result of their numerous benefits, including cheaper cost, more flexibility, remote monitoring, and ease of operation^1^. The rise of big data technologies in recent years has nonetheless progressively increased the amount of data transmitted across networks, placing enormous pressure on the constrained network capacity. The event-triggered control (ETC) approach successfully addresses this issue. Because of this, the subject of ETC in NCSs has received a lot of attention lately (some relevant findings are described in^2^ and its references).

ETC’s key benefit is its ability to conserve network traffic while maintaining the system’s essential functionality. According to the methodology, several issues with conventional NCSs were expanded to the event-triggered variants. For instance, using the state and fixed threshold event-triggered version known as general mixed ETC. For instance, Li et al.^3^ investigated $H_\infty$ control in a networked linear parameter changing system. The issues with quantized stabilization for event-triggered NCSs with data packet losses and plant uncertainties were examined in paper^4^. An event-triggered networked T-S fuzzy system with interval time-varying delays was researched in the publication^5^.

An enhanced ETC of a network-based T-S fuzzy system was suggested in the publication^6^ for the tracking control of NCSs. Numerous papers have been published recently that focus on the relative ETC as a particular example of general ETC, including $H_\infty$ control for networked Markov jump system^7^, networked singular system with quantization^8^, and discrete-time nonlinear networked singular system^9^. The reference model was introduced in^10^, and in contrast to the conventional approach with the fixed triggered condition, the new adaptive ETC’s triggered condition was controlled by the state error.

In addition, the output-based general mixed ETC was expanded to include output feedback control (OFC) combined non-uniform sampling and $H_\infty$ in^11^. In addition, $H_\infty$ control for nonlinear NCSs, which was modeled by the parallel distributed compensation (PDC) and non-PDC fuzzy control rules, were solved in^12,13^. A novel output-based ETC with a state-dependent threshold, which differs from the mixed ETC with a constant threshold, was introduced in^14^ and addressed the issue of $L_\infty$ control co-design for NCSs with delay and external disturbances. Additionally, the fuzzy filter of the networked T-S fuzzy time-delay system and the $H_\infty$ OFC of Markov jump systems with measured output quantization were explored in^15,16^ for the output-based ETC without threshold. The issues of OFC with an event-triggered architecture of NCSs and distributed NCSs were investigated in^17,18^ by setting the scalar triggered parameter to zero. Numerous additional brand-new ETC algorithms have also been developed, including a revolutionary mode-dependent event-triggering scheme^19^, dynamic event-triggered control (DETC)^20^, decentralized ETC^21^, and adaptive ETC incorporating input and output information^22^.

Additionally, some outstanding findings on the ETC of networked predictive systems were made. For instance, given the asynchronous coordination between subsystems, a mixed time/event-triggered dual-mode distributed predictive control algorithm was developed in paper^23^. Paper^24^ provided innovative solutions to the unsynchronized problem for NCSs with delay using a model-based periodic ETC configuration. The model-based event-triggered predictive control problem for NCSs with delay and data dropout, were discussed in papers^25,26^, respectively. In^27^, a unique Lyapunov-based ETC design was provided that took packet dropouts for NPC by the switching system into account. However, there are not many DETC for networked predictive control systems, and there are no published findings about time-varying gain.

In this paper, we consider the stability and stabilization problems for a class of discrete-time NCSs based on DETC. The networked closed-loop system is established by using the networked delay compensation method and time-delay system approach. The disturbance of the model and random round-trip time (RTT) delay are also included. Motivated by the paper^28^, a dynamic event-triggered network delay compensation control (DET-NDCC) strategy with time-varying gains is presented. Compared to the fixed gain of traditional NPC, this method has less conservatism. On the sensor side, an DETC exists to determine whether the signal is transmitted. On the controller side, a time-varying gains feedback controller is designed to generate a sequence of control signals. On the actuator side, a networked delay compensator (NDC) is set to select the suitable control signal for the plant depending on the networked-induced delay. The main contributions of this article are given:

- By integrating the network delay compensation approach and ETC, a dynamic event-triggered network delay compensation control strategy is proposed. The method combines the merits of reducing the networked bandwidth occupation and compensating for network delay actively.
- Based on the predictive control, delay-dependent state feedback controllers with time-varying gains are designed. Which have less conservatism than the fixed gain predictive controller^29^.
- The stability and stabilization problems of the dynamic event-triggered NCSs with and without external disturbance are discussed. The $H_\infty$ performance of the system under biased noise is guaranteed.

The rest of the article is organized as follows. “Problem formulation” states the problem formulations. “Main results” gives the stability analysis and controller design. “Robust control of NPC” shows the $H_\infty$ performance based on the proposed method. Simulations are shown in “Simulation and experiment results”. A discussion is shown in “Discussion”, Finally, “Conclusion” presents a conclusion to the paper.

## Problem formulation

Considering the following discrete-time system:

1 $$\begin{aligned} x(k+1)= & {} Ax(k)+Bu(k) + E\omega (k)\nonumber \\ y(k)= & {} Cx(k)\nonumber \\ z(k)= & {} Dx(k) \end{aligned}$$

where $x(k)\in R^n$, $y(k)\in R^q$, and $u(k)\in R^m$ are the state vector, output vector and input vector, respectively. $\omega (k)\in R^m$ is the disturbance input. *A*, *B*, *C*, *D* and *E* are the constant matrices of appropriate dimensions.

**Figure 1:**
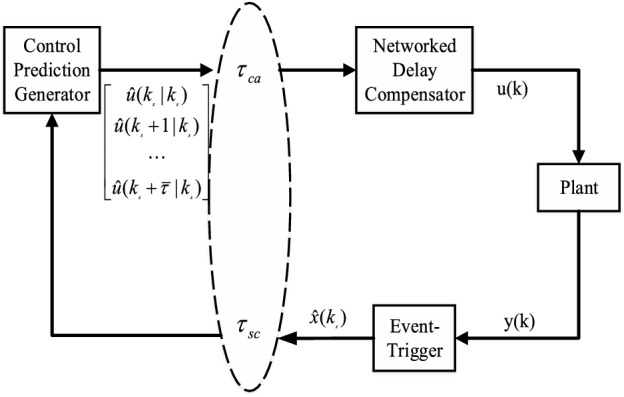
ET-NDCC scheme.

The structure of the NCSs is shown in Fig. 1. On the sensor side, the DETM is added to the system. It is assumed that the state data $x(k_s )$ is transmitted successfully at the time $k_{s}$, the following condition is introduced^28^

2 $$\begin{aligned} \theta (k) + \beta \left( \mu x^T(k_s +r)\Omega x(k_s +r) - [x(k_s +r)-x(k_s )]\Omega [x(k_s +r)-x(k_s)] \right) \le 0 \end{aligned}$$

where $r = \{1, 2, \ldots , N \}$. $0<\mu <1$ is a given scalar parameter. and $\theta (k)$ is determined by

3 $$\begin{aligned} \theta (k+1) = \theta (k) + \left( [x(k_s +r)-x(k_s )]\Omega [x(k_s +r)-x(k_s)] - \mu x^T(k_s +r)\Omega x(k_s +r)\right) \end{aligned}$$

where $\beta >1$, $\theta (0) >0$. Supposing that the first state *x*(0) is transmitted successfully. When the above condition is satisfied, then the state is transmitted, otherwise, it cannot be sent. The system executes the last data and then generates a new state that is verified whether it satisfies the condition (2).

The sequence of states which satisfy the above condition are packed and sent to the controller

4 $$\begin{aligned} \Xi _{k_s } =\left[ {{\begin{array}{*{20}c} {x(k_1 )} &{} {x(k_2 )} &{} \cdots &{} {x(k_s )} \\ \end{array} }} \right] \end{aligned}$$

With the traditional state feedback controller $u(k_s )=Kx(k_s )$, where the gain *K* is fixed for all the time. In consideration of the time variability of networked-induced delay, a more reasonable control law is designed^28^

5 $$\begin{aligned} u(k_s )=K(\tau _k)x(k_s ) \end{aligned}$$

where, the delay $\tau _k=\{0, 1, 2\ldots , {\bar{\tau }}\}$, the feedback gain $K(\tau _k)$ is switched depend on networked-induced delay.

Depending on the different delays, there exists a sequence control signal as follows:

6 $$\begin{aligned} {\hat{u}}(k_s )=K_{i} x(k_s ),\qquad i=\{0, 1,2, \ldots , {\bar{\tau }}\} \end{aligned}$$

where the ${\bar{\tau }}$ is the upper bound of the time-delay. We can select the suitable control signal based on the delay $\tau _k$ at any time $k_{s}$ .The control signals were packed and transmitted to the actuator side in the form as

7 $$\begin{aligned} U_{k_s } =\left[ {{\begin{array}{*{20}c} {{\hat{u}}(k_s )} &{} {{\hat{u}}(k_s +1)} &{} \cdots &{} {\hat{u}(k_s +{\bar{\tau }})} \\ \end{array} }} \right] \end{aligned}$$

On the actuator side, the NDC chooses the suitable control signal depending on the networked-induced delay. The relationship between the delay $\tau _k$, the current time *k* and the event-triggered time $k_s$ is $\tau _k =k-k_s$. And the predictive controller is

8 $$\begin{aligned} u(k)=u(k\vert k-\tau _k )=u(k_s +\tau _k \vert k_s ) \end{aligned}$$

Then, when the $\omega (k)=0$, the closed-loop system is

9 $$\begin{aligned} x(k+1)=Ax(k)+BK(\tau _k )x(k_s ) \end{aligned}$$

Referring to the article^30^, the relationship between triggering time and networked delay is divided into the following two cases.

Case 1: If $k_i +1+{\bar{\tau }}\ge k_{i+1} +\tau _{k_{i+1} }$, define a function $\tau (k)$ as

10 $$\begin{aligned} \tau (k)=k-k_s,\quad k\in [k_s +\tau _{k_s },k_{s+1} +\tau _{k_{s+1} } ) \end{aligned}$$

It follows from Eq. (9) that

11 $$\begin{aligned} \tau _{k_s } \le \tau (k)\le (k_{s+1} -k_s )+\tau _{k_{s+1} } \le 1+{\bar{\tau }} \end{aligned}$$

Case 2: If $k_i +1+{\bar{\tau }}<k_{i+1} +\tau _{k_{i+1} }$, consider the following intervals $\left[ {k_s +\tau _{k_s } ,k_s +{\bar{\tau }}} \right]$and $\left[ {k_s +{\bar{\tau }}+l,k_s +{\bar{\tau }}+l+1} \right]$, where $l \in Z+$ satisfying $l \ge 1$. There must be a positive integer *d* satisfying

12 $$\begin{aligned} k_i +d+{\bar{\tau }}<k_{s+1} +\tau _{k_{s+1} } \le k_i +d+{\bar{\tau }}+1 \end{aligned}$$

Define a function $\tau (k)$ as

13 $$\begin{aligned} \tau (k)=\left\{ {{\begin{array}{*{20}c} {k-k_s,\quad \quad \quad k\in \textrm{Z}_0 } \\ {k-k_s -l,\quad \;\;k\in \textrm{Z}_i } \\ {k-k_s -d_m,\quad k\in \textrm{Z}_{d_m } } \\ \end{array} }} \right. \end{aligned}$$

where $$\left\{ {\begin{array}{l} {\textrm{Z}}_0 =[k_s +\tau _{k_s } ,k_s +{\bar{\tau }}+1) \\ {\textrm{Z}}_i =[k_s +i+{\bar{\tau }},k_s +i+{\bar{\tau }}+1) \\ {\textrm{Z}}_{d_m } =[k_s +d_m +{\bar{\tau }},k_{s+1} +\tau _{k_{s+1} } ) \\ \end{array}} \right.$$

Then it can be easily shown that

14 $$\begin{aligned} \left\{ {\begin{array}{l} \tau _k \le \tau (k)\le 1+{\bar{\tau }}{\mathop {=}\limits ^{\Delta }} d_m,\quad \,k\in \textrm{Z}_0 \\ \tau _k \le {\bar{\tau }}\le \tau (k)\le d_m,\quad \quad k\in \textrm{Z}_i \\ \tau _k \le {\bar{\tau }}\le \tau (k)\le d_m,\quad \quad k\in \textrm{Z}_{d_m } \\ \end{array}} \right. \end{aligned}$$

In Case 1, define $e(k)=0$. In Case 2, define

15 $$\begin{aligned} e(k)=\left\{ {\begin{array}{l} 0,\quad \quad \quad \quad \quad \quad \quad \;\,k\in \textrm{Z}_0 \\ x(k_s )-x(k_s +r),\quad \;\;k\in \textrm{Z}_i \\ x(k_s )-x(k_s +d_m ),\quad k\in \textrm{Z}_{d_m } \\ \end{array}} \right. \end{aligned}$$

Combine the *e*(*k*) and the event-triggered condition (2), we have

16 $$\begin{aligned} e^T(k)\Omega e(k)\le \mu x^T(k-\tau (k))\Omega x(k-\tau (k)) \end{aligned}$$

After that, for $k\in [k_s +\tau _{k_s } ,k_{s+1} +\tau _{k_{s+1} } )$, closed-loop system (1) without disturbance can be further rewritten as

17 $$\begin{aligned} x(k+1)=Ax(k)+ BK(\tau _k )x(k-\tau (k)) + BK(\tau _k )e(k) \end{aligned}$$

The purpose of this paper is to propose appropriate control strategies to make the above systems still operate stably under the influence of delay and interference, and the frequency of data transmission can be reduced.

### Remark 1

As we all know, Zeno behavior refers to an event triggering an infinite number of times for a finite period of time, which can occur in the study of event triggering in continuous-time systems. But in a discrete-time control system, the worst-case scenario for event triggering is that data is sent at every moment k, i.e. time triggering. Therefore, even if the event trigger of the discrete-time system occurs in the Zeno behavior, the worst case will disable its ability to reduce the frequency of data transmission, but it will not affect the stability of the system.

## Main results

In this section, the stability and controller design are addressed. Selecting a switched Lyapunov–Krasovskii function is to to prove the stability of the system.

### Stability analysis

Firstly, the stability problem of closed-loop NPC systems without disturbance is addressed.

#### Theorem 1

*For given parameter* $\mu>$ 0 *and gains* $K_i$, *system* (17) *is asymptotically stable if there exist real matrices* $P_i >0$, $Q_i >0$, $R_i >0$, $S_i >0$, $\Omega _i>$ 0 *and* $X_i$, $Y_i$ *with appropriate dimensions such that*

18 $$\begin{aligned}{} & {} \Xi _i =\left[ {{\begin{array}{*{20}c} {\Phi _i +\xi _i +\xi _i^T +iS_i } &{} *&{} *\\ {\Psi _{21} } &{} {-\lambda P_i } &{} *\\ {\Psi _{31} } &{} 0 &{} {-d_m R_i } \\ \end{array} }} \right] <0, \end{aligned}$$

19 $$\begin{aligned}{} & {} \left[ {{\begin{array}{*{20}c} {S_i } &{} {X_i } \\ *&{} {\frac{1}{\lambda }R_i } \\ \end{array} }} \right] \ge 0,\quad \left[ {{\begin{array}{*{20}c} {S_i } &{} {Y_i } \\ *&{} {\frac{1}{\lambda }R_i } \\ \end{array} }} \right] \ge 0 \end{aligned}$$

20 $$\begin{aligned}{} & {} P_j \le \lambda P_i \quad Q_j \le \lambda Q_i \quad R_j \le \lambda R_i \end{aligned}$$

where $$\Phi _i =\left[ {{\begin{array}{*{20}c} {\lambda (Q_i -P_i )} &{} *&{} *&{} *\\ 0 &{} {\mu \Omega } &{} *&{} *\\ 0 &{} 0 &{} {-Q_i } &{} *\\ 0 &{} 0 &{} 0 &{} {-\Omega } \\ \end{array} }} \right]$$, $$\xi _i =\left[ {{\begin{array}{*{20}c} {X_i } &{} {Y_i -X_i } &{} {-Y_i } &{} 0 \\ \end{array} }} \right]$$, $$\Psi _{21} =\left[ {{\begin{array}{*{20}c} {P_i A} &{} {P_i BK_i } &{} 0 &{} {P_i BK_i } \\ \end{array} }} \right]$$, $$\Psi _{31} =\left[ {{\begin{array}{*{20}c} {R_i (A-I)} &{} {R_i BK_i } &{} 0 &{} {R_i BK_i } \\ \end{array} }} \right]$$, and $$K_i=K(\tau _k)$$.

#### Proof

Construct the following Lyapunov–Krasovskii function as

21 $$\begin{aligned} V_i (k)= & {} V_i^1 (k)+V_i^2 (k)+V_i^3 (k) + \theta (k) =x^T(k)P_i x(k)+\sum \limits _{l=k-d_m }^{k-1} {x^T(l)Q_i x(l)} \nonumber \\{} & {} + \sum \limits _{s=-d_m }^{-1} {\sum \limits _{l=k+s}^{k-1} {\delta ^T(l)R_i \delta (l)} } + \theta (k) \end{aligned}$$

where $$\delta (l)=x(l+1)-x(l)$$.

From Eq. (16), for all $k\in \left( k_s, k_{s+1} \right)$,

22 $$\begin{aligned} \theta (k) + \beta \left( \mu x^T(k-\tau (k))\Omega x(k-\tau (k)) - e^T(k)\Omega e(k) \right) > 0 \end{aligned}$$

If $\beta >1$, we have

23 $$\begin{aligned} \mu x^T(k-\tau (k))\Omega x(k-\tau (k)) - e^T(k)\Omega e(k) > - \frac{1}{\beta }\theta (k) \end{aligned}$$

Then, it can be obtained that

24 $$\begin{aligned} \theta (k+1) > \left( 1 - \frac{1}{\beta }\right) \theta (k) \end{aligned}$$

and $\theta (0)>0$, we have $\theta (k)>0$

25 $$\begin{aligned} \Delta \theta (k) = \theta (k+1) - \theta (k) = \mu x^T(k-\tau (k))\Omega x(k-\tau (k)) - e^T(k)\Omega e(k) \end{aligned}$$

Define $\Delta V_i(k)=V_{\tau _{k+1} } (k+1)-V_i(k)$, Along the solution of system (17), we obtain that

26 $$\begin{aligned} \Delta V_i (k)= \, & {} \Delta V_i^1 (k)+\Delta V_i^2 (k)+\Delta V_i^3 (k) + \Delta \theta (k) \nonumber \\= \, & {} x^T(k+1)P_{\tau _{k+1} } x(k+1)-x^T(k)P_i x(k) + x^T(k)Q_{\tau _{k+1} } x(k)\nonumber \\{} & {} -x^T(k-d_m )Q_i x(k-d_m ) +d_m \delta ^T(k)R_i \delta (k)-\sum \limits _{l=k-d_m }^{k-1} {\delta ^T(l)R_{\tau _{k+1} } \delta (l)} \nonumber \\= \, & {} \left( {Ax(k)+BK(\tau _k )x(k-\tau (k))+ BK(\tau _k )e(k)} \right) ^TP_{\tau _{k+1} } \left( {Ax(k) +BK(\tau _k )x(k-\tau (k))+BK(\tau _k )e(k)} \right) \nonumber \\{} & {} -x^T(k)P_ix(k) + x^T(k)Q_{\tau _{k+1} } x(k) \nonumber \\{} & {} -x^T(k-d_m )Q_i x(k-d_m ) + d_m \delta ^T(k)R_i \delta (k)\nonumber \\{} & {} -\sum \limits _{l=k-d_m }^{k-1} {\delta ^T(l)R_{\tau _{k+1} } \delta (l)} + \mu x^T(k-\tau (k))\Omega x(k-\tau (k)) - e^T(k)\Omega e(k) \end{aligned}$$

Based on the free-weighting matrix approach^31^, it can be seen that

27 $$\begin{aligned}{} & {} x(k)-x(k-d_m )-\sum \limits _{l=k-d_m }^{k-1} {\delta (l)} =0 \end{aligned}$$

28 $$\begin{aligned}{} & {} i\eta _1^T (k)S_i \eta _1 (k)-\sum \limits _{l=k-d}^{k-1} {\eta _1^T (l)S_i \eta _1 (l)} =0 \end{aligned}$$

Combing the Eqs. (25), (26), (27), (28), we have

29 $$\begin{aligned} \Delta V_i (k)= & {} \Delta V_i^1 (k)+\Delta V_i^2 (k)+\Delta V_i^3 (k) + \Delta \theta (k) \nonumber \\\le & {} \Delta V_i^1 (k)+\Delta V_i^2 (k)+\Delta V_i^3 (k) \nonumber \\{} & {} + 2 \eta ^T _1 X_i \left[ x(k)-x(k-\tau (k) )-\sum \limits _{l=k-\tau (k) }^{k-1} {\delta (l)} \right] + 2 \eta ^T _1 Y_i \left[ x(k-\tau (k) )-x(k-d_)-\sum \limits _{l=k-d_m }^{k-\tau (k)} {\delta (l)} \right] \nonumber \\{} & {} + d_m\eta _1^T (k)S_i \eta _1 (k)-\sum \limits _{l=k-d}^{k-1} {\eta _1^T (l)S_i \eta _1 (l)} + \mu x^T(k-\tau (k))\Omega x(k-\tau (k)) - e^T(k)\Omega e(k) \end{aligned}$$

where $$\eta _1 =\left[ {{\begin{array}{*{20}c} {x^T(k)} &{} {x^T(k-d(k))} &{} {x^T(k-d_m )} &{} {e (k)} \\ \end{array} }} \right] ^T$$.

Further, let $P_j \le \lambda P_i, Q_j \le \lambda Q_i, R_j \le \lambda R_i$, the formula (29) can be simplified to

30 $$\begin{aligned} \begin{array}{l} \Delta V_i (k)\le \eta _1^T (k)\Xi _i \eta _1 (k) \!-\! \sum \limits _{l=k-\tau (k)}^{k-1} {\eta _2^T (k)} \left[ \! {{\begin{array}{*{20}c} {S_i } &{} {X_i } \\ *&{} {\frac{1}{\lambda }R_i } \\ \end{array} }} \! \right] \eta _2 (k) -\sum \limits _{l=k-d_m }^{k-\tau (k)} {\eta _2^T (k)} \left[ \!{{\begin{array}{*{20}c} {S_i } &{} {Y_i } \\ *&{} {\frac{1}{\lambda }R_i } \\ \end{array} }} \! \right] \eta _2 (k) \\ \end{array} \end{aligned}$$

where $$\eta _2 =\left[ {{\begin{array}{*{20}c} {\eta _1^T } &{} {\delta ^T(l)} \\ \end{array} }} \right] ^T$$.

The condition of theorem one can be made by Shure’s complement theorem. If condition (18–20) is met, then $\Delta V_i (k)<0$, i.e. system (17) is asymptotically stable. The proof is completed. $\square$

#### Remark 2

The Lyapunov–Krasovskii function introduces a time-delay term, which makes it less conservative in analyzing the stability of time-delay systems. The value of the Barrier Lyapunov function (BLF) tends to infinity as the function variable approaches the constraint boundary, as shown in the figure. This means that while the BLF remains bounded by the designed controller, the function variables remain within the constraint boundary, i.e. the constraint is satisfied. This makes BLF more advantageous in dealing with systems with state constraints.

### Controller design

Based on Theorem 1, the delay-dependent controller design method for the system (17) can be easily obtained.

#### Theorem 2

*For given parameters* $\lambda >1$, $\mu >0$ *and* $d_m$, *under the event-triggering condition* (2, 3), *the system in* (17) *is stabilizable, if there exist matrices* ${\bar{Q}}_i >0$, $W_i >0$, $V_i >0$, ${\bar{S}}_i >0$, ${\bar{\Omega }}_i >0$, ${\bar{X}}_i$, ${\bar{Y}}_i$, ${\bar{K}}_i$ *with appropriate dimensions satisfying the following LMIs*:

31 $$\begin{aligned}{} & {} \left[ {{\begin{array}{*{20}c} {{\bar{\Phi }}_i +{\bar{\xi }}_i +{\bar{\xi }}_i^T +i{\bar{S}}_i } &{} *&{} *\\ {{\bar{\Psi }}_{21} } &{} {-\lambda W_i } &{} *\\ {{\bar{\Psi }}_{31} } &{} 0 &{} {-d_m V_i } \\ \end{array} }} \right] <0 \end{aligned}$$

32 $$\begin{aligned}{} & {} \left[ \!\! {{\begin{array}{*{20}c} {S_i } &{} {X_i } \\ *&{} {\frac{1}{\lambda }(2W_i -V_i )} \\ \end{array} }}\!\! \right] \ge 0, \quad \left[ \!\! {{\begin{array}{*{20}c} {S_i } &{} {Y_i } \\ *&{} {\frac{1}{\lambda }(2W_i -V_i )} \\ \end{array} }}\!\! \right] \ge 0, \end{aligned}$$

33 $$\begin{aligned}{} & {} W_j \le \lambda W_i, \quad V_j \le \lambda V_i \end{aligned}$$

*where* $${\bar{\Phi }}_i =\left[ {{\begin{array}{*{20}c} {\lambda ({\bar{Q}}_i -W_i )} &{} *&{} *&{} *\\ 0 &{} {\mu {\bar{\Omega }}_i } &{} *&{} *\\ 0 &{} 0 &{} {-{\bar{Q}}_i } &{} *\\ 0 &{} 0 &{} 0 &{} {-{\bar{\Omega }}_i } \\ \end{array} }} \right] , {\bar{\xi }}_i =\left[ {{\begin{array}{*{20}c} {{\bar{X}}_i } &{} {{\bar{Y}}_i -{\bar{X}}_i } &{} {-{\bar{Y}}_i } &{} 0 \\ \end{array} }} \right]$$, $${\bar{\Psi }}_{21} =\left[ {{\begin{array}{*{20}c} {AW_i } &{} {B{\bar{K}}_i } &{} 0 &{} {B{\bar{K}}_i } \\ \end{array} }} \right] , {\bar{\Psi }}_{31} =\left[ {{\begin{array}{*{20}c} {(A-I)W_i } &{} {B{\bar{K}}_i } &{} 0 &{} {B{\bar{K}}_i } \\ \end{array} }} \right]$$. *In addition, by solving LMI and matrix transformation, the switched controller gains matrices are given by* $K_i ={\bar{K}}_i W_i^{-1}$.

#### Proof

Pre- and post-multiplying Eqs. (18), (19) with $\Lambda _1 =\text{ diag }\{P_i^{-1} ,P_i^{-1} ,P_i^{-1} ,P_i^{-1} ,P_i^{-1} ,R_i^{-1} \}$, $\Lambda _2 =\text{ diag }\{P_i^{-1} ,P_i^{-1} , P_i^{-1}, P_i^{-1} \},$ respectively. and defining some new variables as $W_i =P_i^{-1}$, $V_i =R_i^{-1}$, ${\bar{Q}}_i =P_i^{-1} Q_i P_i^{-1}$, ${\bar{R}}_i =P_i^{-1} R_i P_i^{-1}$,${\bar{\Omega }}_i =P_i^{-1} \Omega _i P_i^{-1}$, ${\bar{S}}_i =\Lambda _2 S_i \Lambda _2$, ${\bar{\xi }}_i =\Lambda _2 \xi _i \Lambda _2$. The Eq. (30) can be obtained. The proof is completed. $\square$

## Robust control of NPC

Now, we are in a position to solve the problems of stability of NPC system with disturbance and $H_\infty$ controller design of NPC system with event-triggered mechanism. The networked closed-loop system with disturbance is obtained that

34 $$\begin{aligned} x(k+1)=Ax(k)+ BK(\tau _k )x(k-\tau (k))+ BK(\tau _k )e(k)+ E\omega (k) \end{aligned}$$

### Stability analysis

#### Theorem 3

*For given* $\gamma$, $\mu$ *and matrices* $K_i$, *the system is stable with an* $H_\infty$ *norm bound if there exist matrices* $P_{i}>0, Q_i>0, R_i>0, S_i>0, \Omega _i > 0$ *and* $X_{i}, Y_i$ *with appropriate dimensions such that*

35 $$\begin{aligned}{} & {} \Theta _i \!=\!\left[ {{\begin{array}{*{20}c} {\Phi _i +\xi _i +\xi _i^T +iS_i } &{} *&{} *&{} *&{} *\\ {\varphi _{21} } &{} {-\lambda P_i } &{} *&{} *&{} *\\ {\varphi _{31} } &{} 0 &{} {-\gamma ^2I} &{} *&{} *\\ {\varphi _{41} } &{} 0 &{} {R_i E} &{} {-d_m R_i } &{} *\\ {\varphi _{51} } &{} 0 &{} 0 &{} 0 &{} {-I} \\ \end{array} }} \right] \! < \! 0 \end{aligned}$$

36 $$\begin{aligned}{} & {} \left[ {{\begin{array}{*{20}c} {S_i } &{} {X_i } \\ *&{} {\frac{1}{\lambda }R_i } \\ \end{array} }} \right] \ge 0, \quad \left[ {{\begin{array}{*{20}c} {S_i } &{} {Y_i } \\ *&{} {\frac{1}{\lambda }R_i } \\ \end{array} }} \right] \ge 0 \end{aligned}$$

where $$\varphi _{21} =\Psi _{21} , \quad \varphi _{31} =\left[ {{\begin{array}{*{20}c} {\left( {P_i E} \right) ^T} &{} 0 &{} 0 &{} 0 \\ \end{array} }} \right] , \quad \varphi _{41} =\Psi _{31} , \quad \varphi _{51} =\left[ {{\begin{array}{*{20}c} D &{} 0 &{} 0 &{} 0 \\ \end{array} }} \right]$$.

#### Proof

For the system (34) with the disturbance vector $\omega (k)$, we considers the following Lyapunov function $\Delta V(k)=\Delta V_i(k) + \Delta \theta (k)$:

37 $$\begin{aligned} \Delta V_i^1 (k)= & {} x^T(k+1)P_{\tau _{k+1} } x(k+1)-x^T(k)P_i x(k) \nonumber \\= & {} \left( {Ax(k) + BK(\tau _k )x(k - \tau (k)) + BK(\tau _k )e(k) + E\omega (k)} \right) ^TP_{\tau _{k+1} } \nonumber \\{} & {} \left( {Ax(k) + BK(\tau _k )x(k - \tau (k)) +BK(\tau _k )e(k) + E\omega (k)} \right) - x^T(k)P_i x(k) \end{aligned}$$

38 $$\begin{aligned} \Delta V_i^2 (k)= & {} x^T(k)Q_{\tau _{k+1} } x(k)-x^T(k-d_m )Q_i x(k-d_m ) \end{aligned}$$

39 $$\begin{aligned} \Delta V_i^3 (k)= & {} d_m \delta ^T(k)R_i \delta (k)-\sum \limits _{l=k-d_m }^{k-1} {\delta ^T(l)R_{\tau _{k+1} } \delta (l)} \end{aligned}$$

where $$\delta (k) = \left( {A - I} \right) x(k) + BK(\tau _k )x(k - \tau (k)) + BK(\tau _k )e(k) + E\omega (k)$$

It follows Theorem 1 that

40 $$\begin{aligned} \Delta V(k) +z^T(k)z(k)-\gamma ^2\omega ^T(k)\omega (k)<0 \end{aligned}$$

when $\omega (k)=0$, $\Delta V<0$ which implies that the closed-loop system (33) is asymptotically stable Combing the Eqs. (25), (37–40), we have

41 $$\begin{aligned} \begin{array}{l} \Delta V (k)\le \eta _1^T (k)\Theta _i \eta _1 (k)-\sum \limits _{l=k-\tau (k)}^{k-1} {\eta _2^T (k)} \left[ {{\begin{array}{*{20}c} {S_i } &{} {X_i } \\ *&{} {\frac{1}{\lambda }R_i } \\ \end{array} }} \right] \eta _2 (k) -\sum \limits _{l=k-d_m }^{k-\tau (k)} {\eta _2^T (k)} \left[ {{\begin{array}{*{20}c} {S_i } &{} {Y_i } \\ *&{} {\frac{1}{\lambda }R_i } \\ \end{array} }} \right] \eta _2 (k) \\ \end{array} \end{aligned}$$

For all $k = \{1, 2, 3, \ldots \}$, summing Eq. (40) from *k*= 0 to $k =\infty$, it follows that

42 $$\begin{aligned} \sum \limits _{k=0}^\infty {z^T(k)z(k)-} \sum \limits _{k=0}^\infty {\gamma ^2\omega ^T(k)\omega (k)<-} V(\infty ) \end{aligned}$$

From $V(\infty )\ge 0$ and inequality (Eq. 41), it can be seen that

43 $$\begin{aligned} \sum \limits _{k=0}^\infty {z^T(k)z(k)<} \sum \limits _{k=0}^\infty {\gamma ^2\omega ^T(k)\omega (k)} \end{aligned}$$

Thus, the *H* $\infty$-norm of the closed-loop system (Eq. 34) is less than $\gamma$. The proof is completed. $\square$

### Controller design

Similar to the Theorem 2, the switched *H* $\infty$ controller gains $K_i$ for system (28) are calculated by the following theorem.

#### Theorem 4

*For given parameters* $\lambda >1$, $\mu >0 \gamma$ *and* $d_m$, *under the event-triggering condition* (2, 3), *the system in* Eq. (34) *is stabilizable, if there exist matrices* ${\bar{Q}}_i >0$, $W_i >0$, $V_i >0$, ${\bar{S}}_i >0$, ${\bar{\Omega }}_i >0$, ${\bar{X}}_i$, ${\bar{Y}}_i$,${\bar{K}}_i$ *with appropriate dimensions satisfying the following LMIs*:

44 $$\begin{aligned}{} & {} \left[ {{\begin{array}{*{20}c} {{\bar{\Phi }}_i +{\bar{\xi }}_i +{\bar{\xi }}_i^T +i{\bar{S}}_i } &{} *&{} *&{} *&{} *\\ {{\bar{\varphi }}_{21} } &{} {-\lambda W_i } &{} *&{} *&{} *\\ {{\bar{\varphi }}_{31} } &{} 0 &{} {-\gamma ^2I} &{} *&{} *\\ {{\bar{\varphi }}_{41} } &{} 0 &{} E &{} {-d_m V_i } &{} *\\ {{\bar{\varphi }}_{51} } &{} 0 &{} 0 &{} 0 &{} {-I} \\ \end{array} }} \right] <0 \end{aligned}$$

45 $$\begin{aligned}{} & {} \left[ {{\begin{array}{*{20}c} {S_i } &{} {X_i } \\ *&{} {\frac{1}{\lambda }(2W_i -V_i )} \\ \end{array} }} \right] \ge 0, \quad \left[ {{\begin{array}{*{20}c} {S_i } &{} {Y_i } \\ *&{} {\frac{1}{\lambda }(2W_i -V_i )} \\ \end{array} }} \right] \ge 0, \end{aligned}$$

where $${\bar{\Phi }}_i =\left[ {{\begin{array}{*{20}c} {\lambda ({\bar{Q}}_i -W_i )} &{} *&{} *&{} *\\ &{} {\mu {\bar{\Omega }}_i } &{} *&{} *\\ &{} 0 &{} {-{\bar{Q}}_i } &{} *\\ &{} 0 &{} 0 &{} {-{\bar{\Omega }}_i } \\ \end{array} }} \right] , {\bar{\xi }}_i =\left[ {{\begin{array}{*{20}c} {{\bar{X}}_i } &{} {{\bar{Y}}_i -{\bar{X}}_i } &{} {-{\bar{Y}}_i } &{} 0 \\ \end{array} }} \right] , {\bar{\varphi }}_{31} =\left[ {{\begin{array}{*{20}c} {E^T} &{} 0 &{} 0 &{} 0 \\ \end{array} }} \right]$$, ${\bar{\varphi }}_{21} ={\bar{\Psi }}_{21}$, $${\bar{\varphi }}_{51} =\left[ {{\begin{array}{*{20}c} {DW_i } &{} 0 &{} 0 &{} 0 \\ \end{array} }} \right]$$, ${\bar{\varphi }}_{41} ={\bar{\Psi }}_{31}$. *In addition, by solving LMI and matrix transformation, the switched controller gains matrices are given by* $K_i ={\bar{K}}_i W_i^{-1}$, $i=\{0,1,\ldots ,{\bar{\tau }}\}$ .

*The proof is similar to the Theorem* 2, *thus omitted*.

## Simulation and experiment results

In this section, two numerical examples are given to verify the effectiveness of the approach we presented.

### Example 1: stabilization of the networked system without disturbance

In this case, a discrete-time linear system without disturbance is considered as the following

$$\begin{aligned} A=\left[ {{\begin{array}{*{20}c} 1&{} {0.1} &{} {-0.0124} &{} {-0.0004} \\ 0 &{} 1 &{} {-0.25} &{} {-0.0124} \\ 0 &{} 0 &{} {0.0619} &{} {0.1021} \\ 0 &{} 0 &{} {1.2502} &{} {0.0619} \\ \end{array} }} \right] , B=\left[ {{\begin{array}{*{20}c} {0.0013} \\ {0.0251} \\ {-0.0013} \\ {-0.0255} \\ \end{array} }} \right] C =\left[ {{\begin{array}{*{20}c} {1} &{} {0} &{} {1} &{} {0} \\ \end{array} }} \right] \end{aligned}$$

**Figure 2:**
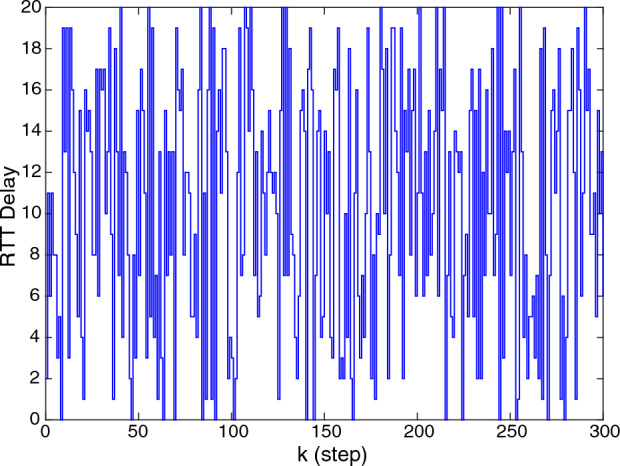
0–20 steps RTT delay.

The parameters mentioned in Theorem 2 are given: $d_m =21,\lambda =1,\mu =0.5, \theta (0)=1, \beta =1$. To solve the Theorem 2 by using LMI, the time-varying gains and the weight matrix $\Omega$ can be obtained as follows

$$\begin{aligned} K_0= & {} \left[ {{\begin{array}{*{20}c} {-0.4182} &{} {-4.5700} &{} {10.2852} &{} {-1.2935} \\ \end{array} }} \right] \quad K_1 =\left[ {{\begin{array}{*{20}c} {-0.9118} &{} {-6.9234} &{} {12.7267} &{} {-1.3184} \\ \end{array} }} \right] \\ K_2= & {} \left[ {{\begin{array}{*{20}c} {-1.2904} &{} {-7.3054} &{} {11.3521} &{} {-1.0508} \\ \end{array} }} \right] \quad K_3 =\left[ {{\begin{array}{*{20}c} {-1.5045} &{} {-7.1984} &{} {9.7771} &{} {-0.7720} \\ \end{array} }} \right] \\ K_4= & {} \left[ {{\begin{array}{*{20}c} {-1.5492} &{} {-6.5036} &{} {8.2041} &{} {-0.6189} \\ \end{array} }} \right] \quad K_5 =\left[ {{\begin{array}{*{20}c} {-1.6552} &{} {-6.4370} &{} {7.2551} &{} {-0.4647} \\ \end{array} }} \right] \\ K_6= & {} \left[ {{\begin{array}{*{20}c} {-1.7207} &{} {-6.0150} &{} {6.3257} &{} {-0.3604} \\ \end{array} }} \right] \quad K_7 =\left[ {{\begin{array}{*{20}c} {-1.7594} &{} {-5.8761} &{} {5.4663} &{} {-0.2627} \\ \end{array} }} \right] \\ K_8= & {} \left[ {{\begin{array}{*{20}c} {-1.7464} &{} {-5.6220} &{} {4.7712} &{} {-0.1855} \\ \end{array} }} \right] \quad K_9 =\left[ {{\begin{array}{*{20}c} {-1.7205} &{} {-5.3558} &{} {4.1906} &{} {-0.1273} \\ \end{array} }} \right] \\ K_{10}= & {} \left[ {{\begin{array}{*{20}c} {-1.6970} &{} {-5.1087} &{} {3.7046} &{} {-0.0787} \\ \end{array} }} \right] \quad K_{11} =\left[ {{\begin{array}{*{20}c} {-1.5701} &{} {-4.8953} &{} {3.3090} &{} {-0.0405} \\ \end{array} }} \right] \\ K_{12}= & {} \left[ {{\begin{array}{*{20}c} {-1.5710} &{} {-4.6538} &{} {2.9730} &{} {-0.0166} \\ \end{array} }} \right] \quad K_{13} =\left[ {{\begin{array}{*{20}c} {-1.3876} &{} {-4.4442} &{} {2.6613} &{} {0.0104} \\ \end{array} }} \right] \\ K_{14}= & {} \left[ {{\begin{array}{*{20}c} {-1.3216} &{} {-4.2497} &{} {2.4016} &{} {0.0326} \\ \end{array} }} \right] \quad K_{15} =\left[ {{\begin{array}{*{20}c} {-1.2637} &{} {-4.0826} &{} {2.1766} &{} {0.0538} \\ \end{array} }} \right] \\ K_{16}= & {} \left[ {{\begin{array}{*{20}c} {-1.1328} &{} {-3.8776} &{} {2.0252} &{} {0.0558} \\ \end{array} }} \right] \quad K_{17} =\left[ {{\begin{array}{*{20}c} {-1.1203} &{} {-3.7168} &{} {1.8447} &{} {0.0707} \\ \end{array} }} \right] \\ K_{18}= & {} \left[ {{\begin{array}{*{20}c} {-0.9853} &{} {-3.5508} &{} {1.7263} &{} {0.0725} \\ \end{array} }} \right] \quad K_{19} =\left[ {{\begin{array}{*{20}c} {-0.9646} &{} {-3.4422} &{} {1.5978} &{} {0.0837} \\ \end{array} }} \right] \\ K_{20}= & {} \left[ {{\begin{array}{*{20}c} {-0.8595} &{} {-3.2692} &{} {1.4981} &{} {0.0812} \\ \end{array} }} \right] \quad \Omega =\left[ {{\begin{array}{*{20}c} {0.0594} &{} {0.0028} &{} {-0.0009} &{} {-0.0000} \\ {0.0028} &{} {0.0620} &{} {-0.0126} &{} {0.0010} \\ {-0.0009} &{} {-0.0126} &{} {0.1493} &{} {-0.0176} \\ {-0.0000} &{} {0.0010} &{} {-0.0176} &{} {0.0453} \\ \end{array} }} \right] \end{aligned}$$

**Figure 3:**
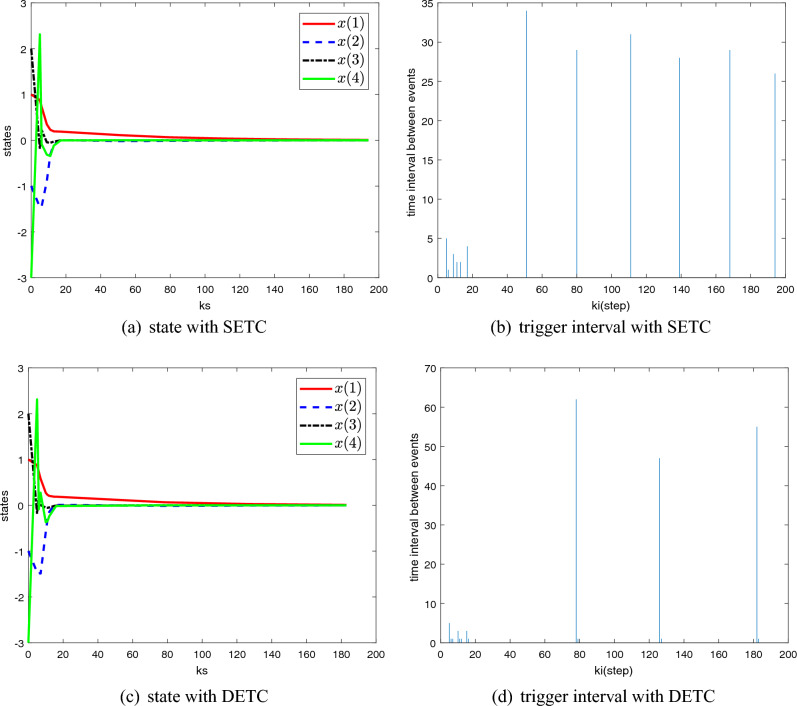
Comparison of state and trigger interval between SETC and DETC.

Considering the 0–20 steps random RTT delay shown in Fig. 2, a comparison of the results of the two event-triggered control methods (SETC and DETC) is shown in Fig. 3. Obviously, with similar stability results, DETC has fewer trigger moments than SETC.

**Figure 4:**
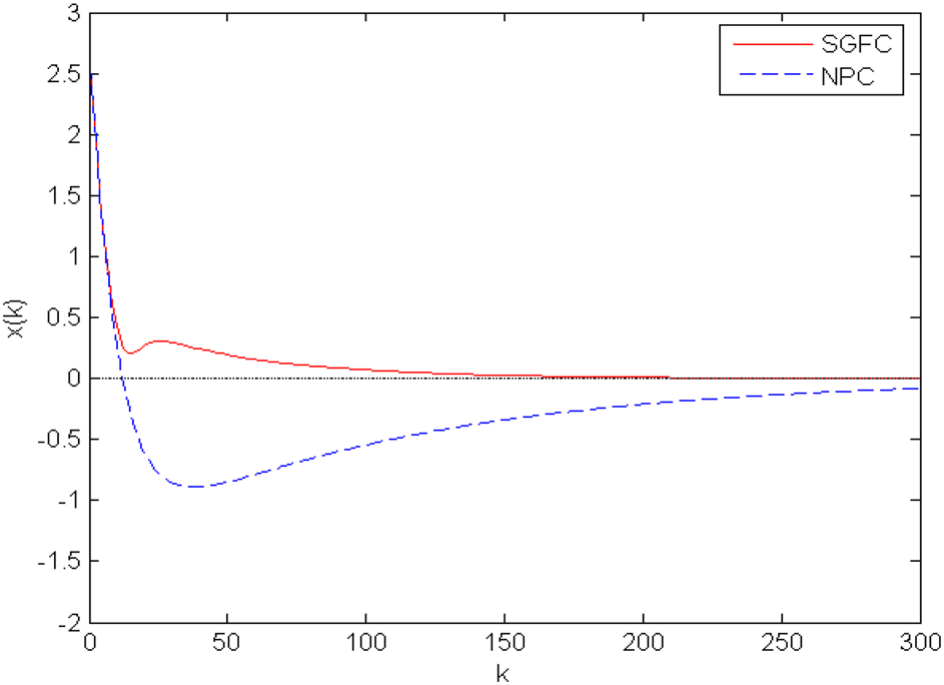
The results of $x_1$ between NPC and our method.

In addition, the comparison of the methods between NPC with fixed gains and time-varying gains is given. The simulation results is shown in Fig. 4. As can be seen from the pictures, even if the system has random delay, the method we proposed can still make the system eventually stable and reduce the frequency of data transmission. In addition, we compare the proposed method with the fixed-gain NPC method, and the results show that our method can converge faster.

### Example 2: stabilization of the networked event-triggered control system with disturbance

In this case, the robust $H_\infty$ control of NCSs: the ETC and NDC approach is verified, the system parameters is following

$$\begin{aligned} A=\left[ {{\begin{array}{*{20}c} {0.85} &{} 0 &{} {0.1} \\ {0.01} &{} {0.96} &{} 0 \\ 0 &{} 0 &{} 1 \\ \end{array} }} \right] , \quad B=\left[ {{\begin{array}{*{20}c} {-0.1} \\ {-0.2} \\ {-0.1} \\ \end{array} }} \right] , \quad E=\left[ {{\begin{array}{*{20}c} 1 \\ 1 \\ 1 \\ \end{array} }} \right] , C=\left[ {{\begin{array}{*{20}c} 1 &{} 1 &{} 1 \\ \end{array} }} \right] , \quad D=\left[ {{\begin{array}{*{20}c} {0.1} &{} {0.1} &{} {0.1} \\ \end{array} }} \right] \end{aligned}$$

The disturbance is set to be $$\omega (k)=\left\{ {{\begin{array}{*{20}c} {sign(sin(k)) ,\quad \quad k<30}\\ {0,\quad \quad \quad \quad \quad \quad \quad k\ge 30 } \\ \end{array} }} \right.$$

**Figure 5:**
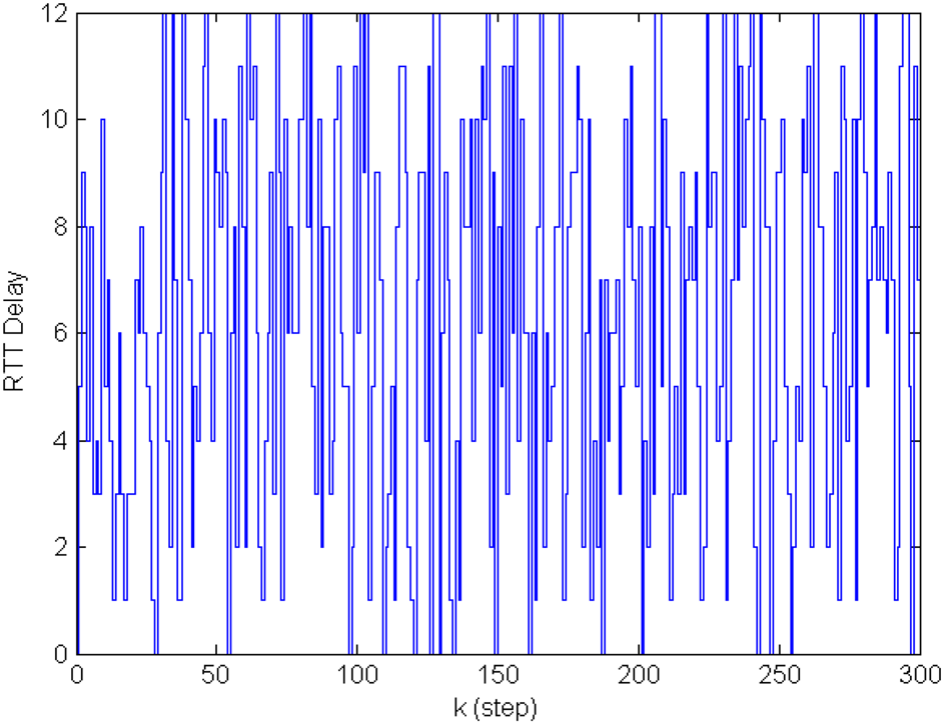
0–12 steps RTT delay.

**Figure 6:**
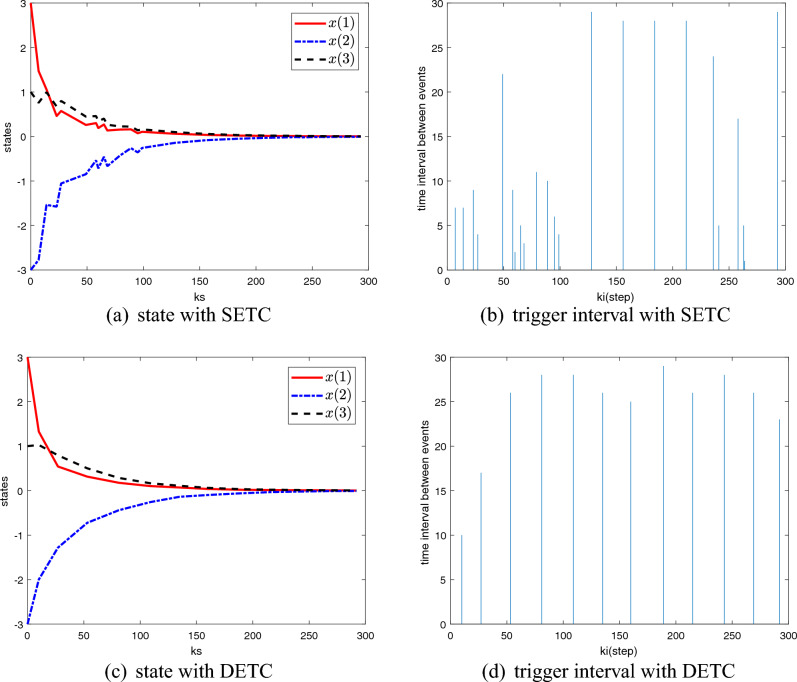
Comparison of state and trigger interval between SETC and DETC with disturbance.

The random delay is shown in the Fig. 5. And the parameters in event-triggered condition (3) and the Theorem 4 are $d_m =12, \lambda =2, \mu =0.5, \gamma =0.01, \theta (0)=0.01, \beta =5$. Then the controller gains and the parameter $\Omega$ can be solved by MATLAB LMI toolbox

$$\begin{aligned} K_0= & {} \left[ {{\begin{array}{*{20}c} {3.2014} &{} {3.2563} &{} {3.3342} \\ \end{array} }} \right] , \quad K_1 =\left[ {{\begin{array}{*{20}c} {1.5060} &{} {1.6461} &{} {1.8564} \\ \end{array} }} \right] , \quad K_2 =\left[ {{\begin{array}{*{20}c} {1.0648} &{} {1.2005} &{} {1.4097} \\ \end{array} }} \right] , \\ K_3= & {} \left[ {{\begin{array}{*{20}c} {0.7520} &{} {0.8549} &{} {1.0152} \\ \end{array} }} \right] , \quad K_4 =\left[ {{\begin{array}{*{20}c} {0.5765} &{} {0.6586} &{} {0.7870} \\ \end{array} }} \right] , \quad K_5 =\left[ {{\begin{array}{*{20}c} {0.4621} &{} {0.5316} &{} {0.6410} \\ \end{array} }} \right] , \\ K_6= & {} \left[ {{\begin{array}{*{20}c} {0.3780} &{} {0.4372} &{} {0.5306} \\ \end{array} }} \right] , \quad K_7 =\left[ {{\begin{array}{*{20}c} {0.3044} &{} {0.3512} &{} {0.4251} \\ \end{array} }} \right] , \quad K_8 =\left[ {{\begin{array}{*{20}c} {0.2568} &{} {0.2987} &{} {0.3652} \\ \end{array} }} \right] , \\ K_9= & {} \left[ {{\begin{array}{*{20}c} {0.2209} &{} {0.2561} &{} {0.3117} \\ \end{array} }} \right] , \quad K_{10} =\left[ {{\begin{array}{*{20}c} {0.1875} &{} {0.2181} &{} {0.2669} \\ \end{array} }} \right] , \quad K_{11} =\left[ {{\begin{array}{*{20}c} {0.1677} &{} {0.1954} &{} {0.2394} \\ \end{array} }} \right] , \\ K_{12}= & {} \left[ {{\begin{array}{*{20}c} {0.1533} &{} {0.1803} &{} {0.2201} \\ \end{array} }} \right] , \quad \Omega =\left[ {{\begin{array}{*{20}c} {0.0174} &{} {0.0115} &{} {0.0123} \\ {0.0115} &{} {0.0199} &{} {0.0145} \\ {0.0123} &{} {0.0145} &{} {0.0230} \\ \end{array} }} \right] . \end{aligned}$$

In the case of disturbance and delay, the system state and event trigger interval are shown in Fig. 6. As can be seen from Figs. 6a–d, the system eventually remains stable despite some jitter under disturbance and delay. And the number of data transfers is reduced, and the DETC method performs better than SETC. The simulation results in this part show that the proposed method can still make the system converge in the presence of delay and disturbance.

## Discussion

This paper combines dynamic event trigger control and networked predictive control methods and presents a delay compensation control scheme based on dynamic event triggering. This solution allows the system to operate stably under the influence of delay and disturbance and reduces data transmission.

Compared with the static event triggering method, the method proposed in this article can further reduce data transmission without affecting system stability. The introduction of event gain avoids the control of fixed gain for better performance. The scheme based on networked predictive control can actively compensate for the delay, which is less conservative than the traditional method.

LMI can only get sufficient conditions to stabilize the system. Secondly, LMI is an optimization tool, but multiple matrices are scaled in the stability analysis, so the result may be a suboptimal solution. In addition, delay-dependent controller gain switching improves control performance, but it is difficult to derive its globally stable conditions. Issues such as less conservative adequacy and overall optimal performance will be the focus of our future research.

## Conclusion

This paper has addressed the DETC problem of discrete-time networked predictive control systems with simultaneous consideration of delays and disturbance. The closed-loop system is obtained by investigating the NDC method, the DETC method, and the feedback time delay-dependent gain method. The DETC method can effectively reduce the network bandwidth resources occupied by data transmission. The random delay has compensated by NDC actively. A dynamic event-triggered network delay compensation control strategy has proposed. Then, the delay-dependent stability conditions have been derived by using the LMI approach. Based on these conditions, the time-varying gain predictive controller has designed. Furthermore, the robust $H_\infty$ control problem of NCSs has discussed. Finally, simulations illustrate the effectiveness of the proposed algorithm.

## Data Availability

All data generated or analysed during this study are included in this published article.
